# Eliminating Enzymatic Interference: How Drying Methods Modulate Non-Enzymatic Browning and Quality Retention in Pre-Inactivated *Artemisia dracunculus* L.

**DOI:** 10.3390/foods15193454

**Published:** 2026-09-27

**Authors:** Yue Cui, Zhen Wang, Mingxi Jia, Bing Li, Muhtar Kadirhaz, Longfei Han, Haixia Han

**Affiliations:** 1 College of Food Science and Pharmacy, Xinjiang Agricultural University, Urumqi 830052, China; 320242506@stu.xjau.edu.cn (Y.C.); 15609973796@163.com (Z.W.); 120240097@xjau.edu.cn (M.J.); muhtar@xjau.edu.cn (M.K.); 320252582@stu.xjau.edu.cn (L.H.); 2 Xinjiang Key Laboratory of Postharvest Fruit Science and Technology, Urumqi 830052, China; 3 College of Modern Industry in Chinese Herbal Medicine, Xinjiang Agricultural University, Urumqi 830052, China; 4 Manas Meion Asafoetida Ecological Technology Co., Ltd., Changji 832200, China; mnaw2026111@163.com

**Keywords:** *Artemisia dracunculus* L., non-enzymatic browning, drying methods, quality characteristics

## Abstract

*Artemisia dracunculus* L. is prone to post-harvest spoilage, and drying often causes browning that reduces color and nutritional quality. Since enzymatic and non-enzymatic browning occur simultaneously and interact, their individual contributions are difficult to isolate. To address this, we used hot-water blanching to strongly inhibit endogenous oxidases (residual PPO/POD activities < 3%), thus largely eliminating enzymatic interference, and investigated non-enzymatic browning under four drying methods: shade drying (SD), vacuum freeze-drying (VFD), microwave drying (MD), and hot-air drying (HAD). We compared browning phenotype, substrate consumption, reaction products, and bioactive retention, and performed correlation analysis and PCA. VFD markedly suppressed non-enzymatic browning, showing the lowest browning degree (0.212 A/g), highest chlorophyll retention (62.17%), and best color stability (L* = 41.19, ΔE = 5.88). Correlation analysis revealed that vitamin C depletion was more closely correlated with browning than the measured Maillard reaction products. Based on these lab-scale results, each drying method offers a distinct reference value: VFD is preferred for high-value products requiring strict browning control; MD suits moderate-quality needs; SD provides a low-cost option for general quality; and HAD fits conventional drying with low quality demands. This work provides a theoretical basis for targeted regulation of non-enzymatic browning during *A. dracunculus* drying.

## 1. Introduction

*Artemisia dracunculus* L. (*A. dracunculus*) has a distinctive aroma and possesses antioxidant and blood lipid-regulating properties, making it highly valuable for industrial development. Fresh *A. dracunculus* is highly susceptible to spoilage after harvest, and drying is a common processing method used to extend its shelf life [1]. However, the browning that occurs during the drying process can impair the raw material’s color and degrade its nutritional and functional components, thereby limiting its potential for further processing.

Browning in plants is primarily divided into enzymatic browning and non-enzymatic browning. Enzymatic browning is catalyzed by endogenous redox enzymes such as polyphenol oxidase (PPO) and peroxidase (POD), and occurs rapidly only during the early stages of drying; non-enzymatic browning involves multiple reaction pathways, including the Maillard reaction, ascorbic acid oxidation, and chlorophyll demagnesium degradation, and occurs continuously throughout the entire drying cycle [2]. *A. dracunculus* possesses high endogenous oxidase activity and contains abundant browning substrates such as phenols, chlorophyll, and reducing sugars. In traditional direct drying processes, enzymatic and non-enzymatic browning occur simultaneously and influence each other, making it extremely difficult to distinguish and elucidate the patterns and regulatory mechanisms of non-enzymatic browning, as well as to precisely control this process. Most existing studies have failed to isolate the interference from enzymatic reactions; in mixed browning systems, it is impossible to analyze the independent effect of drying methods on non-enzymatic browning, making it difficult to clarify the intrinsic relationship between browning substrates, products, and product quality [3].

In recent years, the regulatory effects of different drying techniques on non-enzymatic browning in horticultural and aromatic plants have been extensively explored [4]. Vacuum freeze-drying effectively suppresses the Maillard reaction and ascorbic acid oxidation under low-temperature anaerobic conditions, delivering the lowest browning degree and the best retention of bioactive components [5]. Hot air drying operates in a high-temperature aerobic environment, which markedly accelerates the consumption of reducing sugars and ascorbic acid, causing prominent accumulation of non-enzymatic browning products [6]. Microwave drying sharply cuts down drying time and thus reduces the duration of browning reactions, but local high temperature still triggers partial browning [7]. As a traditional low-energy approach, shade drying has a long processing cycle that enables sufficient progression of non-enzymatic browning.

However, for *A. dracunculus*, most existing studies were conducted under natural conditions with active endogenous oxidases, where enzymatic and non-enzymatic browning are coupled, making it impossible to accurately distinguish the independent regulatory effects of drying methods on non-enzymatic browning pathways [8,9]. To fill this research gap, this study employed hot-water blanching pretreatment to strongly inhibit endogenous oxidase activities and construct a non-enzymatic browning system with minimal PPO/POD-mediated interference for the first time. After verifying the enzyme-inactivating effect [10], four drying methods—shade drying, vacuum freeze-drying, microwave drying, and hot-air drying—were compared. The study systematically investigated the patterns of how different drying methods affect non-enzymatic browning from the perspectives of browning appearance indicators, consumption of browning substrates, accumulation of Maillard reaction products, and retention of active components such as phenolics. Combining correlation analysis and PCA, this study reveals the intrinsic coupling relationships between non-enzymatic browning and various quality indicators, and screens a suitable drying process that balances browning inhibition with the preservation of raw material quality. This research provides a theoretical basis for the targeted regulation of non-enzymatic browning in *A. dracunculus* drying.

## 2. Materials and Methods

### 2.1. Materials

Fresh plant samples were collected from Wenquan County (Xinjiang, China). All samples were taxonomically identified as *Artemisia dracunculus* L. by professional botanists, and healthy, pest-free individuals with uniform growth status were selected for subsequent experiments to reduce experimental heterogeneity. For reagent supplies, 1,1-Diphenyl-2-picrylhydrazyl (DPPH, purity ≥ 98.5%) and 2,2′-azino-bis (3-ethylbenzothiazoline-6-sulfonic acid) diammonium salt (ABTS, purity ≥ 99%) were obtained from Nanjing Jiancheng Bioengineering Institute, (Nanjing, China). HPLC-grade reagents including methanol, acetonitrile, acetic acid, and isopropanol were purchased from Sinopharm Chemical Reagent Co., Ltd. (SCRC), (Shanghai, China) while ammonium acetate was sourced from Sigma-Aldrich (St. Louis, MO, USA). All other analytical-grade reagents, including polyethylene glycol (PEG), polyvinylpyrrolidone (PVP), Triton X-100, catechol, guaiacol, anhydrous ethanol, acetone, trichloroacetic acid, sodium nitrite, aluminum nitrate, sodium hydroxide, sodium carbonate, 3,5-dinitrosalicylic acid, 2,6-dichloroindophenol sodium salt, oxalic acid, ascorbic acid, Folin–Ciocalteu reagent, sulfanilic acid, N-(1-naphthyl)ethylenediamine dihydrochloride, salicylic acid, gallic acid, and rutin, were purchased from Yuanye Bio-Technology Co., Ltd. (Shanghai, China).

### 2.2. Enzyme Inactivation Pretreatment

Fresh *A. dracunculus* with uniform appearance, intact structure and good color was selected, washed, and cut into 4.0 cm segments. Afterwards, 20 g samples were blanched at 100 °C with a solid–liquid ratio of 1:20 (g:mL) for 10, 40, 80, 120, and 160 s, according to a previously reported thermal blanching protocol [11]. After blanching, the samples were immediately cooled to room temperature in an ice-water bath to obtain enzyme-inactivated *A. dracunculus* (EI-AD).

### 2.3. Preparation of Dried Samples

Four commonly used drying methods were applied to the pretreated EI-AD samples, with drying terminated once the dry-base moisture content of the material fell below 10%. All drying treatments adopted the same initial sample mass (20 g) with a uniform spreading thickness of 10 mm.

Prior to the formal experiment, single-factor preliminary tests were conducted to screen key process parameters. Five temperature gradients (50–90 °C) for hot air drying and five power gradients (80–800 W) for microwave drying were set. With drying rate, sensory quality and active component retention as comprehensive evaluation indicators, 80 °C and 440 W were finally determined as the optimal parameters for the two processes, respectively. The parameters of shade drying and vacuum freeze-drying were set according to standard aromatic herb drying protocols and equipment specifications. The specific parameters of each drying method were set as follows:

Shade drying (SD): Samples were dried in a ventilated environment away from direct sunlight, with an air velocity of approximately 0.4 m/s. The total drying time was approximately 24 h.

Vacuum freeze-drying (VFD): Samples were first pre-frozen at −18 °C for 12 h, then dried by vacuum sublimation at −40 °C under a vacuum pressure of 20 Pa using a SCIENTZ-10N/C freeze dryer (Ningbo Scientz Biotechnology Co., Ltd., Ningbo, China). The total drying time was approximately 12 h.

Microwave drying (MD): MD was carried out at a power of 440 W, with the material spread to a uniform thickness of 10 mm, using the BYM-100 microwave vacuum drying oven (Shanghai Boxun Industrial Co., Ltd., Shanghai, China). The total drying time was approximately 120 s.

Hot air drying (HAD): HAD was conducted at 80 °C with a cross-flow air velocity of 1.5 m/s, using a DHG-9140A electric blast drying oven (DHG series; Shanghai Shenxian Constant Temperature Equipment Factory, Shanghai, China). The total drying time was approximately 4.5 h.

Fresh: samples were oven-dried to constant weight at 105 °C, and all values were expressed on a dry-weight (DW) basis.

### 2.4. Enzyme Activity Assays

PPO Activity: 2.0 g EI-AD portions were homogenized in 8 mL ice-cold 100 mM acetate buffer (pH 5.5) supplemented with 1 mM PEG, 4% (*w*/*v*) PVP, and 1% (*w*/*v*) Triton X-100. The homogenate was centrifuged at 12,000× *g* for 30 min at 4 °C, and the supernatant was collected as crude enzyme extract [12]. A 200 µL aliquot of the extract was mixed with 4.0 mL 50 mM acetate buffer (pH 5.5) and 1.0 mL 50 mM catechol, with absorbance at 420 nm recorded continuously for 5 min post-mixing. PPO activity was defined as the enzyme amount causing a 0.01 absorbance increase per hour at 420 nm, expressed as U h^−1^ g^−1^.

POD Activity: POD activity was assayed via guaiacol oxidation in the presence of H_2_O_2_, monitored at 470 nm [12]. A 100 µL extract aliquot was mixed with 3.0 mL 25 mM guaiacol and 200 µL 50 mM H_2_O_2_, with absorbance recorded for 3 min post-initiation. POD activity was defined as the enzyme amount causing a 0.01 absorbance increase per minute at 470 nm, expressed as U min^−1^ g^−1^.

Residual activity for both enzymes was calculated via Equation (1):

(1) Residual enzyme activity (%) = U/U_0_ × 100%

where U and U_0_ represent the enzyme activities after and before blanching, respectively. All determinations were performed in triplicate.

### 2.5. Drying Characteristics

The dry-base moisture content was determined by the direct drying method at 105 °C to constant weight. To characterize the drying behavior of EI-AD during pretreatment, we determined the dry-base moisture content and moisture ratio at different drying times [13].

(2) $$Determination\;of\;\text{dry-base}\;moisture\;content:\;M_{t}=\frac{m_{t}-m_{d}}{m_{d}}$$

where m_t_ is the mass (g) of EI-AD at time t; m_d_ is the dry matter mass (g) of EI-AD.

(3) $$Determination\;of\;moisture\;ratio:\;MR=\frac{M_{t}-M_{e}}{M_{c}-M_{e}}$$

where M_t_, M_c_ and M_e_ are the moisture contents at time t, initially, and at equilibrium, respectively, on a dry basis. Since the equilibrium moisture content of EI-AD was relatively small, Equation (3) can be simplified as Equation (4):

(4) $$MR=\frac{M_{t}}{M_{c}}$$

where M_t_ is the dry-base moisture content at time t, and M_c_ is the initial dry-base moisture content.

(5) $$Determination\;of\;Drying\;Rate:\;DR=\frac{M_{t_{2}}-M_{t_{1}}}{t_{2}-t_{1}}$$

where t_2_ − t_1_, $M_{t_{1}}$ and $M_{t_{2}}$ are the time difference and moisture content (d.b.) at times t_1_ and t_2_, respectively. The drying rate is expressed in g·g^−1^ DM·min^−1^.

### 2.6. Non-Enzymatic Browning and Related Color Changes

(1) Color Parameters

Surface color characteristics including lightness (L*), redness (a*) and yellowness (b*) of EI-AD samples before and after drying were measured using a colorimeter [14]. Total color difference (ΔE) was calculated using Equation (6).

(6) $$\Delta E=\sqrt{{\left(L^{*}-L_{0}\right)}^{2}+{\left(a^{*}-a_{0}\right)}^{2}+{\left(b^{*}-b_{0}\right)}^{2}}$$

where ΔL*, Δa* and Δb* represent the differences in L*, a* and b* values before and after drying, respectively. Samples before drying were used as the control. The total color difference (ΔE) was used to evaluate color retention of EI-AD samples, with a lower ΔE value indicating better color retention and closer appearance to the original sample.

(2) Browning Degree

Browning degree (BD) was determined according to a previous method [15]. Briefly, 0.2 g of ground and sieved EI-AD was extracted with 10 mL of 2% (*v*/*v*) acetic acid for 2 h, stirred for 1 min, and centrifuged at 10,000 rpm for 10 min. Absorbance of the supernatant was measured at 420 nm against 2% acetic acid as blank. BD was calculated using Equation (7).

(7) BD = A_420_ × V/m

where A is the absorbance at 420 nm, V is the volume of extract, and m is the sample mass (g). Triplicate measurements were conducted for each sample.

(3) Total Chlorophyll Content

Chlorophyll content was determined strictly following GB/T 20362-2006 [16]. A 0.5 g sample was extracted with 10 mL of ethanol–acetone mixture (1:1, *v*/*v*) in the dark at room temperature for 5 h. After filtration, absorbances at 645 nm and 663 nm were recorded against the extraction solvent. Total chlorophyll content (Chl) was calculated using Equation (8).

(8) Chl = (8.05 × A_1_ + 20.29 × A_2_) × V/(1000 × m)

where A_1_ and A_2_ are absorbances at 663 nm and 645 nm, respectively; V is the volume of test solution (mL); and m is sample mass (g). All analyses were performed in triplicate.

(4) Microstructure

For microstructure observation, the dried EI-AD samples were cut into small pieces of 10 mm × 5 mm, and sputter-coated with a thin gold layer to enhance electrical conductivity and avoid charging effects during detection. The surface morphology of the samples was observed using an SU8010 scanning electron microscope (Hitachi, Tokyo, Japan), with an accelerating voltage of 5 kV and a magnification of 400×.

### 2.7. Reaction Mechanism of Non-Enzymatic Browning

(1) Reducing sugar content

Reducing sugar content was determined by the 3,5-dinitrosalicylic acid (DNS) method [17]. Ground EI-AD samples (0.5 g) were extracted three times with 10 mL of 80% methanol under ultrasonication at 40 °C for 30 min. After centrifugation at 6000 rpm for 15 min, supernatants were combined and diluted to 50 mL with 80% methanol for further analysis. After reaction with DNS reagent in a boiling water bath, absorbance was measured at 540 nm with glucose as the standard. Content was calculated using Equation (9), with three replicates.

(9) Content (mg/g) = C × S × V/1000 × M

where C is the concentration of the extract (μg/mL), S is the dilution factor, V is the volume of extract (mL), and M is the sample mass (g).

(2) Vitamin C content

Vitamin C content was determined by the 2,6-dichloroindophenol titration method [18]. Sample extracts were prepared in 1% oxalic acid, followed by filtration and centrifugation. Titration was performed to a stable pink endpoint for 15 s. Vitamin C content was calculated using Equation (10), with triplicate determinations.

(10) Vitamin C (mg/g DW) = (V_sample_ − V_blank_) × K × V_t_/V_1_ × V_2_ × m

where V_sample_ and V_blank_ are titration volumes (mL); K = 1 mg/mL is the calibration coefficient; V_t_ is the total extraction volume (mL); V_1_ is the volume of filtrate before centrifugation (mL); V_2_ is the titrated supernatant volume (mL); and m is sample mass (g).

(3) Maillard Fluorescent Compounds

Maillard fluorescent compounds were analyzed according to the method of Chu et al. [19]. A 1 g sample was mixed with 10 mL deionized water, vortexed for 1 min, and diluted to 50 mL. After filtration, 1 mL filtrate was mixed with 1 mL of 40% trichloroacetic acid and centrifuged at 1000 rpm for 10 min. Subsequently, 0.5 mL supernatant was combined with 2.5 mL phosphate buffer. Fluorescence intensity was scanned from 360 to 600 nm at an excitation wavelength of 345 nm, with deionized water as blank. Characteristic fluorescence spectra and peak intensities were recorded.

### 2.8. Determination of Bioactive Components

(1) Total Phenolic and Total Flavonoid Contents

The same extraction procedure as described in Section 2.7 (1) was used. Total flavonoid content (TFC) was determined by the sodium nitrite–aluminum nitrate–sodium hydroxide chromogenic method at 510 nm [20], with rutin as the standard. Total phenolic content (TPC) was measured using the Folin–Ciocalteu method at 765 nm [20], with gallic acid as the standard. Both contents were calculated using Equation (9), with triplicate determinations.

(2) Putative Identification and Qualitative Analysis of polyphenol monomers

For polar metabolites, chromatographic separation was performed on a Waters ACQUITY UPLC BEH Amide column (2.1 mm × 50 mm, 1.7 μm) using a Vanquish UPLC system (Thermo Fisher Scientific, Waltham, MA, USA). Mobile phase A consisted of 25 mmol/L ammonium acetate and 25 mmol/L ammonia in water, and mobile phase B was acetonitrile. The autosampler temperature was maintained at 4 °C, and the injection volume was 2 μL. For nonpolar metabolites, separation was carried out on a Phenomenex Kinetex C18 column (2.1 mm × 50 mm, 2.6 μm). Mobile phase A was water containing 0.01% acetic acid, and mobile phase B was an isopropanol–acetonitrile mixture (1:1, *v*/*v*). The column temperature was 25 °C, autosampler temperature was 4 °C, and injection volume was 2 μL. The retention time range was 0.5–8 min for both modes. Full MS and MS/MS data were acquired using an Orbitrap Exploris 120 mass spectrometer (Thermo Fisher Scientific, Waltham, MA, USA) controlled by Xcalibur 4.4 software (Thermo Fisher Scientific, Waltham, MA, USA). Operating parameters were set as follows: sheath gas flow rate 50 Arb, aux gas flow rate 15 Arb, capillary temperature 320 °C, full MS resolution 60,000, MS/MS resolution 15,000, collision energy SNCE 20/30/40, and spray voltage 3.8 kV (positive mode) or −3.4 kV (negative mode). Mass tolerance: <5 ppm (full MS), <10 ppm (MS/MS). Compounds were putatively annotated via accurate mass, fragments and database matching. Pre-batch calibration and interval QC were applied; data are relative peak responses (Counts/CPS) without absolute quantitation.

### 2.9. Antioxidant Activities

EI-AD extracts used for antioxidant assays were prepared according to Section 2.7 (1) and diluted to 5 mg/mL prior to analysis.

(1) DPPH Radical Scavenging Activity

DPPH scavenging activity was measured according to Bonilla-Loor et al. [21]. Absorbance was recorded at 517 nm. Scavenging rate was calculated using Equation (11).

(11) DPPH scavenging rate (%) = [1 − (A_1_ − A_2_/A_0_)] × 100%

where A_1_ is the absorbance of the sample with DPPH, A_2_ is the background absorbance of the sample alone, and A_0_ is the absorbance of the DPPH blank control.

(2) ABTS^+^ Radical Scavenging Activity

ABTS^+^ scavenging activity was measured following Bonilla-Loor et al. [21]. Absorbance was recorded at 734 nm. The scavenging rate was calculated using Equation (12).

(12) ABTS^+^ scavenging rate (%) = [1 − (B_1_ − B_2_/B_0_)] × 100%

where B_1_ is the absorbance of the sample with ABTS^+^, B_2_ is the background absorbance of the sample alone, and B_0_ is the absorbance of the ABTS^+^ blank control.

(3) Hydroxyl Radical Scavenging Activity

Hydroxyl radical scavenging activity was analyzed according to Bonilla-Loor et al. [21]. Absorbance was determined at 510 nm. The scavenging rate was calculated using Equation (13)

(13) OH^•^ scavenging rate (%) = [1 − (C_1_ − C_2_/C_0_)] × 100%

where C_1_ is the absorbance of the complete reaction system, C_2_ is the background absorbance without H_2_O_2_, and C_0_ is the absorbance of the blank control without sample.

### 2.10. Statistical Analysis

All measurements adopted a hierarchical replication design: three independent plant batches served as biological replicates, with independent drying runs for each method per batch, and all assays were performed in triplicate as analytical replicates. For one-way ANOVA, Pearson correlation and PCA, the mean of each independent drying run was treated as an independent observation; analytical replicates were only used for error reduction and not counted as independent observations in statistical inference. SPSS 26.0 (IBM, Armonk, NY, USA) software was used for statistical analysis, in which one-way analysis of variance (ANOVA) and Duncan’s multiple range test were performed at the 95% confidence level. For kinetic datasets, nonlinear regression was performed using Origin 2024: first-order model for inactivation curves, Page model for drying curves; rate constants were compared to assess overall treatment differences. A correlation heatmap was constructed based on Pearson correlation analysis, and a PCA score plot was drawn to explore the differences among samples and the correlation rule among indicators. All variables were z-score standardized prior to PCA to eliminate unit and range bias. GraphPad Prism 10.1.2 (GraphPad Software, San Diego, CA, USA) and Origin 2024 software (OriginLab Corporation, Northampton, MA, USA) were used to plot the experimental charts.

## 3. Results and Discussion

### 3.1. Validation of the Effect of Pre-Enzyme Inactivation

Polyphenol oxidase (PPO) and peroxidase (POD) are the key enzymes mediating enzymatic browning in fruits and vegetables, and their activities depend on the stable spatial conformation of proteins. High-temperature blanching can achieve irreversible inactivation by destroying the secondary bonds of enzyme proteins and leading to conformational unfolding [10]. In this study, hot-water blanching was adopted for enzyme inactivation, aiming to completely block the enzymatic browning pathway before drying, so as to provide a reliable experimental basis for the subsequent focus on the mechanism of non-enzymatic browning. The results shown in Figure 1 indicated that PPO and POD activities decreased rapidly with blanching time, and were well fitted by the first-order kinetic model. After 80 s of treatment, the relative activities of the two enzymes both decreased to below 3%, losing the ability to catalyze enzymatic browning [22]; further extension of the treatment time did not significantly improve the inhibition of enzyme activity; therefore, 80 s was determined as the appropriate enzyme inactivation condition. This pretreatment largely suppressed the interference caused by PPO/POD-mediated enzymatic reactions at the source, ensuring that the color deterioration, chlorophyll degradation, browning product accumulation and quality changes in EI-AD observed during the subsequent drying process were predominantly attributable to non-enzymatic browning pathways such as Maillard reaction and VC oxidation [23].

### 3.2. Drying Rates of Different Drying Methods

Figure 2 depicts the drying kinetic characteristics of EI-AD samples under four drying methods. As shown in Figure 2a, the moisture ratio decreased continuously with drying time in all groups, with significant differences in dehydration efficiency. MD exhibited the fastest drying rate, achieving complete dehydration within approximately 120 s. HAD and VFD showed moderate drying speeds, while SD required the longest drying cycle of up to 24 h. As presented in Figure 2b, the drying rate curves of all groups showed a single-peak trend of first increasing then decreasing, and the peak values were consistent with the overall dehydration efficiency of each method. This pattern indicated that the drying process was dominated by the falling-rate period, with no obvious constant-rate stage. Kinetic fitting showed the Page model best fitted all drying curves, with the drying rate constant following the order MD > HAD > VFD > SD. These differences in drying kinetics arise from distinct heat and mass transfer mechanisms. MD achieves rapid dehydration via volumetric dielectric heating driven by internal vapor pressure. HAD relies on convective heat transfer and outward liquid water diffusion. VFD proceeds via low-temperature vacuum sublimation with relatively high mass transfer resistance. SD has the slowest rate due to the weak driving force of natural evaporation [24].

### 3.3. Color Changes

As shown in Figure 3, the samples in the VFD group had the color closest to the fresh samples, presenting a bright green color and plump appearance. The MD group showed a dark green color with slight browning, while the HAD and SD groups exhibited obvious dark brown color with significant leaf shrinkage. This pattern of appearance changes was highly consistent with the results of L*, ΔE, and browning degree in Table 1, indicating that chlorophyll retention was closely related to the browning degree of the samples [25].

The L* value reflects the lightness of the sample; a lower L* value indicates a darker sample color and a more severe browning degree [26]. As shown in Table 1, the L* value of the VFD group showed no significant difference from that of the fresh samples, showing the best lightness retention. The SD group had the lowest L* value, with the most severe lightness loss and the darkest color. The L* values of the MD and HAD groups were significantly lower than those of the fresh samples, falling between the two groups mentioned above. The ΔE value reflects the degree of color deviation between the sample and the fresh sample, and a higher value indicates a greater color difference [27]. The VFD group had the lowest ΔE value, with the smallest color difference from the fresh samples and the best color retention effect. The SD group had the highest ΔE value, with the largest color deviation from the fresh samples, which was consistent with the visual observation of the samples [25].

### 3.4. Browning Degree

Browning degree can serve as an important indicator for evaluating the degree of non-enzymatic browning during the drying process, and its value reflects the accumulation of brown products generated via pathways such as Maillard reaction, ascorbic acid degradation, and polyphenol oxidation [26]. As shown in Table 1, the VFD group had the lowest browning degree; the SD group had the highest browning degree; the browning degrees of the MD and HAD groups were significantly higher than that of the VFD group, with their values falling between the two groups mentioned above. The variation trend of browning degree among all groups was highly consistent with the color results, which can intuitively reflect the extent of non-enzymatic browning under different drying methods [25].

### 3.5. Chlorophyll Content

As shown in Table 1, the VFD group had the highest chlorophyll retention rate, which was significantly higher than that of the other drying groups; the SD and HAD groups had the lowest chlorophyll retention rates, with no significant difference between the two. Chlorophyll degradation is an important cause of the green color loss and color darkening of the samples. During the drying process, chlorophyll is prone to undergo pheophytinization to form brown derivatives such as pheophytin, thereby reducing the greenness of the samples and darkening their color [28]. The high-temperature environment in the MD and HAD groups can significantly accelerate the degradation and transformation of chlorophyll, leading to rapid loss of green color; the low-temperature vacuum condition in the VFD group can effectively inhibit chlorophyll degradation, thus achieving a good green color retention [7]. Although the temperature in the SD group was relatively low, the drying cycle was excessively long, and chlorophyll underwent continuous oxidative degradation, resulting in a final chlorophyll retention rate similar to that of the HAD group.

### 3.6. Effects of Different Drying Methods on Microstructure

Scanning electron microscopy (SEM) can intuitively characterize the damage to the cell and stomatal structure of EI-AD caused by different drying methods, providing a microscopic mechanistic explanation for the subsequent differences in macroscopic texture and rehydration performance [29]. As shown in Figure 3, the microstructural integrity of EI-AD under the four drying methods presented a significant gradient change, with the overall performance ranked as VFD > MD > SD > HAD. The VFD group had intact cell structure, with only slight shrinkage of stomata; vacuum freeze-drying maximally preserved the natural structure via moisture sublimation. The MD group maintained high cell integrity, with only minor stomatal damage and slight shrinkage, as the short-time high temperature did not cause large-area structural damage. The SD group showed decreased cell integrity, with damaged stomata and local collapse, and the long-cycle drying induced progressive structural degradation. The HAD group exhibited severe cell shrinkage and adhesion, with complete collapse of stomata, and the high-temperature rapid dehydration led to severe rupture of cell walls.

### 3.7. Reducing Sugar Content

Reducing sugars are the core substrates of the Maillard reaction, and their contents directly determine the reaction rate of non-enzymatic browning. The determination of reducing sugars can reveal the browning regulation mechanism of EI-AD from the substrate level [30]. As shown in Figure 4a, fresh samples contained 1.6 mg/g reducing sugar (DW basis). Among the four drying methods, the content followed: VFD > MD > SD > HAD. The low-temperature vacuum environment of the VFD group greatly inhibited the consumption of reducing sugars by the Maillard reaction, and meanwhile, the formation of ice crystals promoted the hydrolysis of some bound polysaccharides, thus leading to the significantly highest reducing sugar content. The rapid drying of the MD group could terminate the continuous oxidation and reaction consumption of reducing sugars, but local high temperature caused a small amount of thermal degradation, so the content ranked second. The SD group had a long drying cycle, and reducing sugars were consumed through slow oxidation and continuous Maillard reaction, resulting in a medium level of content. The long-term high-temperature aerobic environment of the HAD group caused dual consumption of reducing sugars: thermal decomposition and Maillard reaction consumption to a certain extent, leading to the lowest content.

### 3.8. Vitamin C

Vitamin C is a typical heat-sensitive, oxidation-prone active component in plants and also an important natural antioxidant. Changes in its content not only directly reflect the retention effect of different drying methods on heat-sensitive nutrients but also indirectly reflect the oxidative stress level and non-enzymatic browning process of the samples [31]. As shown in Figure 4b, fresh samples contained 2.6 mg/g DW VC. Among the four drying methods, the content followed: VFD > MD > SD > HAD. The low-temperature vacuum environment of the VFD group could maximally inhibit the thermal degradation and oxidation reaction of VC, thus leading to its significantly highest content. The rapid microwave drying of the MD group could reduce water activity in a short time, reducing the continuous oxidation of VC; however, the local high temperature generated by microwave still caused thermal decomposition of part of VC, so the content was lower than that of the VFD group. The SD group had a long room-temperature drying cycle, and VC was easily oxidized and degraded after long-term exposure to air, resulting in a medium level of content. The HAD group was in a high-temperature aerobic environment for a long time, which significantly aggravated the thermal decomposition and oxidation reaction of VC, leading to the lowest content.

### 3.9. Maillard Reaction Products

The Maillard reaction is a non-enzymatic browning reaction that occurs between free amino groups and reducing sugars, and the generated melanoidins are the core color-developing substances of non-enzymatic browning. Stable characteristic fluorescent products are produced during the reaction, so fluorescence intensity is a classic index for evaluating the process and extent of the Maillard reaction [25,32]. As shown in Figure 5a, the fresh group had extremely low fluorescence intensity with no obvious characteristic fluorescence peak, confirming that this characteristic fluorescence signal only originated from the Maillard reaction products generated during the drying process.

The fluorescence intensity of each group followed the order of SD > HAD > MD > VFD, indicating that the extent of the Maillard reaction varied under different drying conditions. The process of the Maillard reaction is mainly regulated by drying temperature and processing time [33]. The SD group had the longest drying cycle, allowing the Maillard reaction to proceed continuously with sufficient accumulation of fluorescent products, thus resulting in the highest fluorescence intensity. Although the high temperature in the HAD and MD groups accelerated the reaction rate, their processing time was short, leading to limited product accumulation; their fluorescence intensities were similar and significantly lower than that of the SD group. The low-temperature vacuum environment in the VFD group significantly inhibited the Maillard reaction, with the least amount of fluorescent products generated, hence the lowest fluorescence intensity. However, the weak correlation between fluorescent products and browning degree suggests that the Maillard reaction was not the dominant pathway here.

### 3.10. Profiles of Bioactive Components

#### 3.10.1. Total Polyphenol Contents (TPC) and Total Flavonoid Contents (TFC)

Different drying methods significantly affected the retention of active components (TPC and TFC) in EI-AD. TPC was expressed as mg gallic acid equivalent/g dry weight, and TFC as mg rutin equivalent/g dry weight, with significant inter-group differences shown in Figure 5b. Fresh samples contained 3.4 mg GAE/g DW TPC and 0.88 mg RE/g DW TFC. Among the four drying methods, VFD exhibited the highest values for both TPC and TFC, followed by MD, while HAD showed the lowest. The VFD group adopted a low-temperature vacuum environment, which could significantly reduce the thermal loss of heat-sensitive components, so the TPC and TFC were significantly higher than those of all other groups [34]. The MD group had a fast drying rate, which could effectively shorten the exposure time of components to air; however, local high temperature easily caused the degradation of both polyphenols and flavonoids, resulting in both TPC and TFC being slightly lower than those of the VFD group [35]. The SD group had a long drying time, with sufficient contact between the samples and air, leading to relatively obvious loss of active components, and the TPC and TFC were at a medium level [36]. The HAD group was affected by long-term high temperature, with the most significant component degradation, and the TPC and TFC were the lowest [37].

#### 3.10.2. Phenolic Acid Compounds

Phenolic monomers were putatively annotated by HPLC-MS, and all data were presented as relative peak responses in Counts/CPS. As shown in Table 2 and Figure 6, the nine phenolic acid compounds showed a consistent trend: the VFD group had the optimal relative response intensity, followed by the MD and SD groups successively, with the HAD group showing the lowest intensity. Among them, eight phenolic acids, including chlorogenic acid, ferulic acid, 4-hydroxycinnamic acid, 3,5-dicaffeoylquinic acid, salicylic acid, benzoic acid, and caffeic acid, exhibited the highest response in the VFD group; only vanillin had the highest response in the MD group. Salicylic acid showed comparable response values across all four drying methods, indicating similar stability. These results aligned with phenolic acid peak abundance profiles in total ion chromatograms acquired under three chromatographic modes: reversed-phase positive ion mode, reversed-phase negative ion mode, and Amide column negative ion mode. In terms of the mechanism of action, the low-temperature vacuum environment of VFD could effectively block the thermal degradation and oxidation of phenolic acids, thus achieving the best relative response performance for most phenolic acids [35]. The rapid drying of MD could inhibit continuous oxidation, and the microwave thermal effect could promote the release of bound vanillin from cell walls, which explained why vanillin had the highest response in the MD group. However, local high temperature still caused the degradation of some heat-sensitive phenolic acids. The SD group had mild room-temperature drying conditions, but the long drying cycle led to more oxidative loss; the long-term high temperature of HAD accelerated the thermal decomposition and oxidation of phenolic acids, resulting in the poorest overall relative response performance [36].

#### 3.10.3. Flavonoid Compounds

As shown in Table 2 and Figure 6, the eight flavonoid compounds showed a highly similar overall response trend: the response intensities of the VFD group and the MD group were significantly higher than those of the HAD group, and the SD group had the lowest response among all drying methods. Among them, four flavonoids, including hyperoside, isookanin, rutin, and okanin, had the highest response in the VFD group, and the response values of rutin in the VFD and MD groups were similar; the other four flavonoids, including hesperetin, pyrocatechol, naringenin, and kaempferol, had the highest response in the MD group. The above results were in good agreement with the peak abundance characteristics of flavonoids shown in Figure 6. In terms of the mechanism of action, flavonoid compounds have high oxidation sensitivity. The low-temperature vacuum environment of VFD could effectively inhibit their oxidative degradation, achieving the best relative response performance for heat-sensitive flavonoid glycosides [35]. The rapid drying of MD greatly shortened the contact time between flavonoids and oxygen, and the damage to flavonoid structure caused by microwave heating was far less than that of HAD, so most flavonoid aglycones had a higher relative response level in the MD group. The long-term high temperature of HAD accelerated the thermal degradation and oxidation of flavonoids; the SD group had the longest drying cycle, leading to continuous oxidation reaction, which made it the group with the poorest relative response performance of flavonoid compounds among all drying methods [36,37].

### 3.11. Antioxidant Activity

Antioxidant capacity is an important index for evaluating the physiological activity and quality stability of plant materials, and can reflect the overall ability of samples to scavenge free radicals and inhibit oxidation reactions. In this study, the scavenging rates of DPPH, ABTS^+^, and OH^−^ were used as indexes to explore the effects of different drying methods on the antioxidant properties of EI-AD. As shown in Figure 7, among the four drying methods, the comprehensive antioxidant capacity followed the order: VFD > MD > HAD > SD. Notably, the VFD samples exhibited even higher antioxidant activity than fresh samples, which may be attributed to three factors: inactivation of endogenous oxidative enzymes by ultra-low temperature and vacuum, release of bound antioxidant components caused by cell structure disruption during freezing, and inhibition of non-enzymatic oxidation under vacuum and low-temperature conditions. Antioxidant capacity is closely related to non-enzymatic browning, and the retention rate of polyphenols directly affects the reduction capacity and browning inhibition effect of the samples [38]. The VFD group retained polyphenols to the greatest extent, with the optimal antioxidant capacity. The MD group had short-term high temperature, and the antioxidant capacity was still relatively high. The polyphenol loss in the SD and HAD groups was significant, which further promoted non-enzymatic browning [39].

### 3.12. Mechanistic Analysis of Non-Enzymatic Browning and Comprehensive Evaluation of Drying Methods

In contrast to previous mixed browning systems, endogenous PPO and POD (residual activity < 3%) were strongly inhibited before drying in this study, so observed browning and quality changes were predominantly attributable to non-enzymatic reactions, greatly improving the accuracy of pathway correlation analysis. Pearson correlation and PCA were performed to elucidate the regulatory mechanism of non-enzymatic browning and evaluate the comprehensive quality of samples under different drying methods.

As shown in Figure 8a, browning degree (BD) had extremely significant negative correlations with lightness (L*), yellowness (b*), reducing sugar (RS), vitamin C (VC), total polyphenols, total flavonoids, chlorophyll and antioxidant activities, and positive correlations with redness (a*) and total color difference (ΔE). Correlations between Maillard reaction products (MR) and BD were weak. Although SD had the highest MR accumulation, which resulted from the combined effect of comprehensive drying conditions, the correlation between MR and browning was weaker than that between VC depletion and browning. This result indicated that VC depletion was more strongly correlated with non-enzymatic browning than the measured Maillard reaction products. Given the largely suppressed enzymatic interference, VC depletion was closely associated with browning occurrence, but a causal role cannot be established from correlation alone. The direct causal role of VC oxidation remains to be verified by targeted detection of its degradation products. As shown in Figure 8b, the first two principal components explained 92.2% of the total variance, effectively covering the main quality differences among samples. The four drying groups were clearly separated along both PC1 and PC2 axes. PC1 loadings were negative for browning degree and color difference, and positive for vitamin C, phenolics, flavonoids and antioxidant activity, reflecting overall quality retention. Drying methods significantly affected comprehensive quality attributes, with effects driven by combined conditions (temperature, duration, pressure, oxygen exposure, heat transfer) rather than any single parameter. Positive PC1 groups (represented by VFD) had less browning and higher component retention; negative groups (SD and HAD) showed the opposite.

A limitation of this study is that hot-water blanching pretreatment itself may cause partial losses of VC, reducing sugars, phenolics, and chlorophyll, as well as tissue structural changes prior to drying. Therefore, the samples, which received no blanching, served only as raw material reference baselines (on a dry-weight basis) and were not statistically compared with the dried samples. Since all drying groups underwent identical blanching, the observed differences in browning and quality among them can be unequivocally attributed to the drying conditions. Future studies may include a blanched-but-undried control to further distinguish the effects of pretreatment from those of drying.

## 4. Conclusions

In this study, a non-enzymatic browning system with strongly suppressed PPO/POD activities was established in *A. dracunculus* by hot-water blanching pretreatment, with residual activities of both enzymes below 3%, which largely eliminated the interference of PPO/POD-mediated enzymatic browning.

Correlation analysis showed that VC depletion was more closely correlated with non-enzymatic browning than the measured Maillard reaction products under the test conditions. VFD exhibited the most favorable performance in terms of the measured physicochemical indicators, with the lowest browning degree, optimal color retention, and highest retention of bioactive components and antioxidant activity, followed by MD. HAD and SD showed relatively inferior quality performance.

These findings provide a preliminary theoretical reference for the regulation of non-enzymatic browning and the selection of drying processes for *A. dracunculus* at the laboratory batch scale. Given that energy consumption, processing cost, scale-up feasibility, retention of aroma volatiles and sensory quality were not evaluated in this study, further pilot-scale validation is required for industrial application.

## Figures and Tables

**Figure 1:**
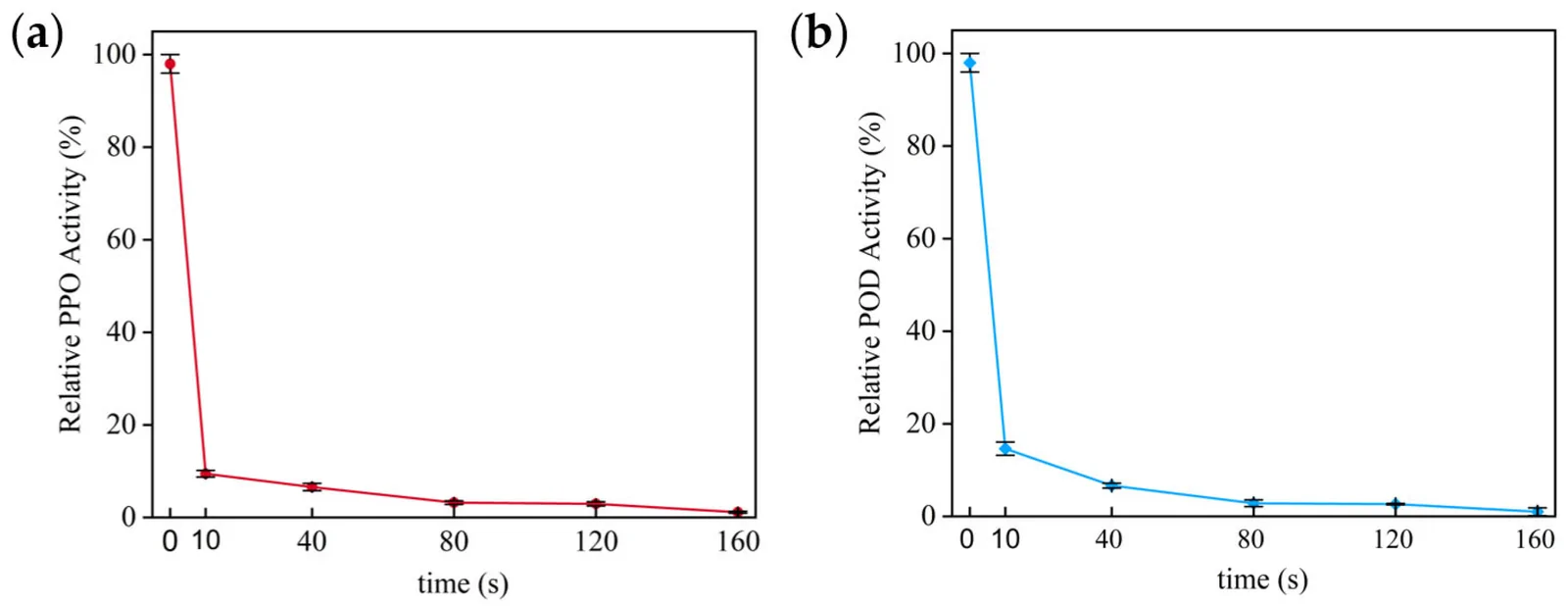
Effect of inactivation time on the enzymatic activity of EI-AD. (**a**) PPO enzyme activity; (**b**) POD enzyme activity. Data are mean ± standard error; curves fitted by first-order kinetic model.

**Figure 2:**
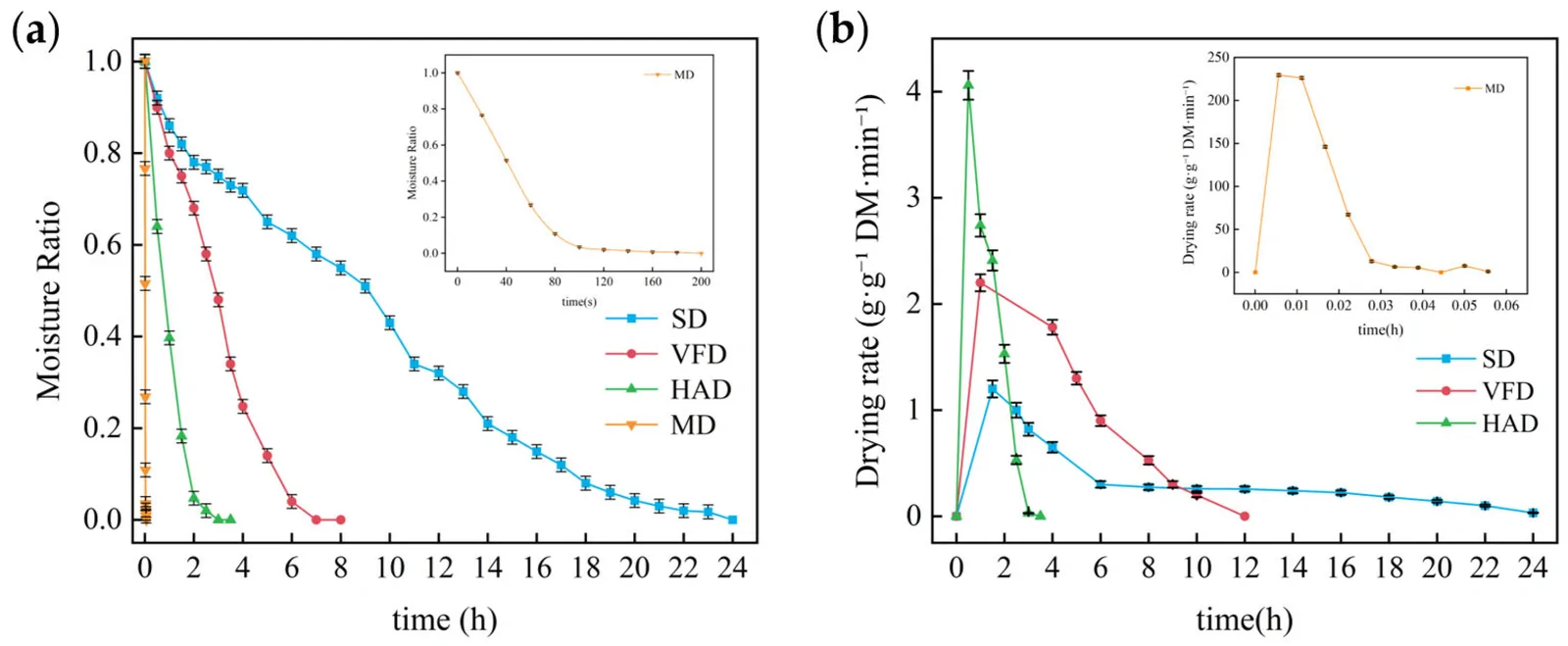
Drying kinetic characteristics of EI-AD under different drying methods. (**a**) Moisture ratio; (**b**) Drying rate. Insets: enlarged MD kinetic curves. Data are mean ± standard error; moisture ratio curves fitted by Page model.

**Figure 3:**
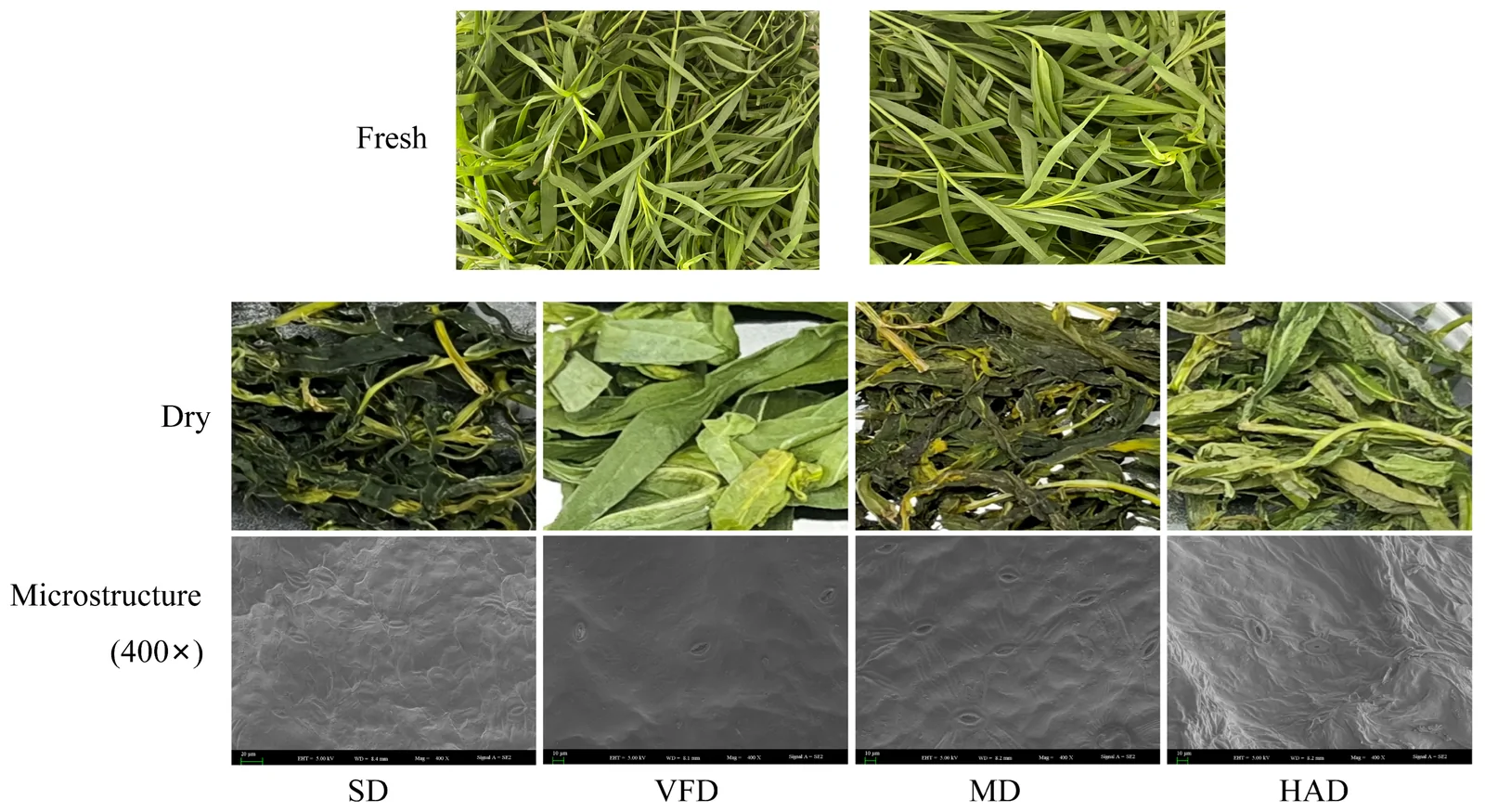
Surface morphology of EI-AD under different drying methods and microstructural surface morphology (400×).

**Figure 4:**
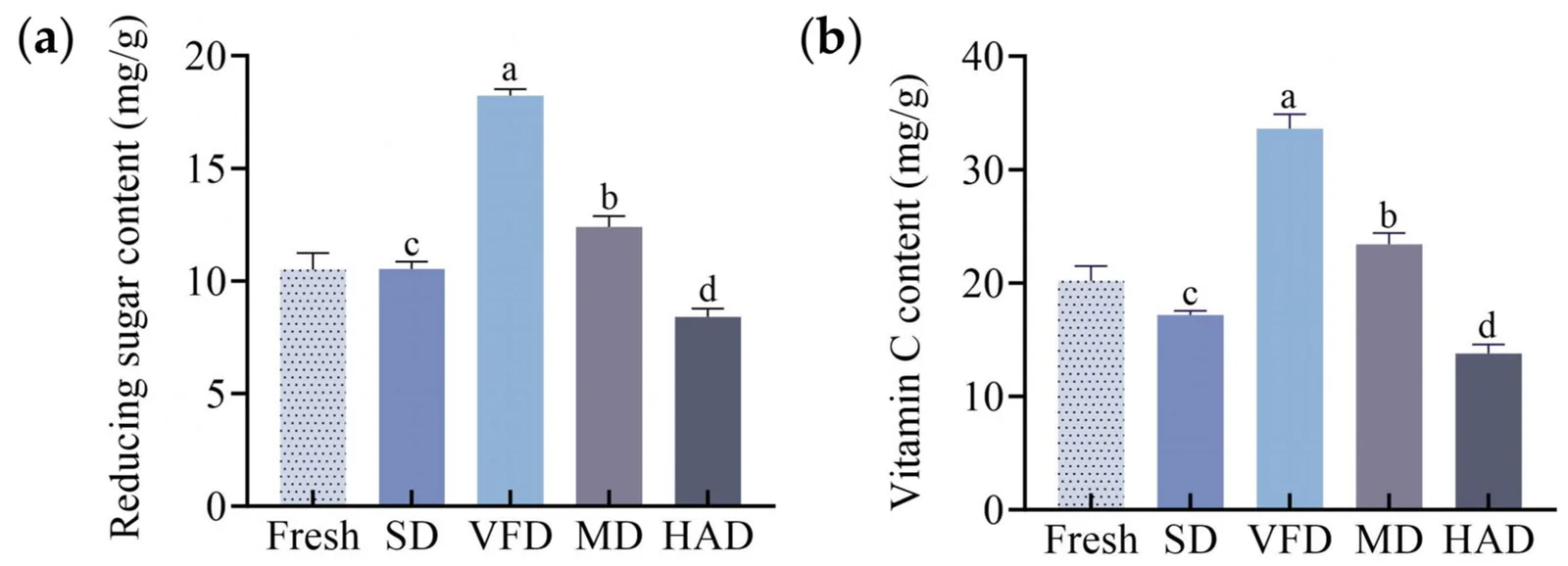
Effects of different drying methods on reducing sugar content and vitamin C content in EI-AD. (**a**) Reducing sugar content; (**b**) Vitamin C content. The fresh sample value (converted to DW basis) is shown as a reference baseline and was not included in the statistical comparison among drying treatments. Statistical comparisons are performed across groups on the horizontal axis. According to Duncan’s test, different lowercase letters indicate significant differences (*p* < 0.05).

**Figure 5:**
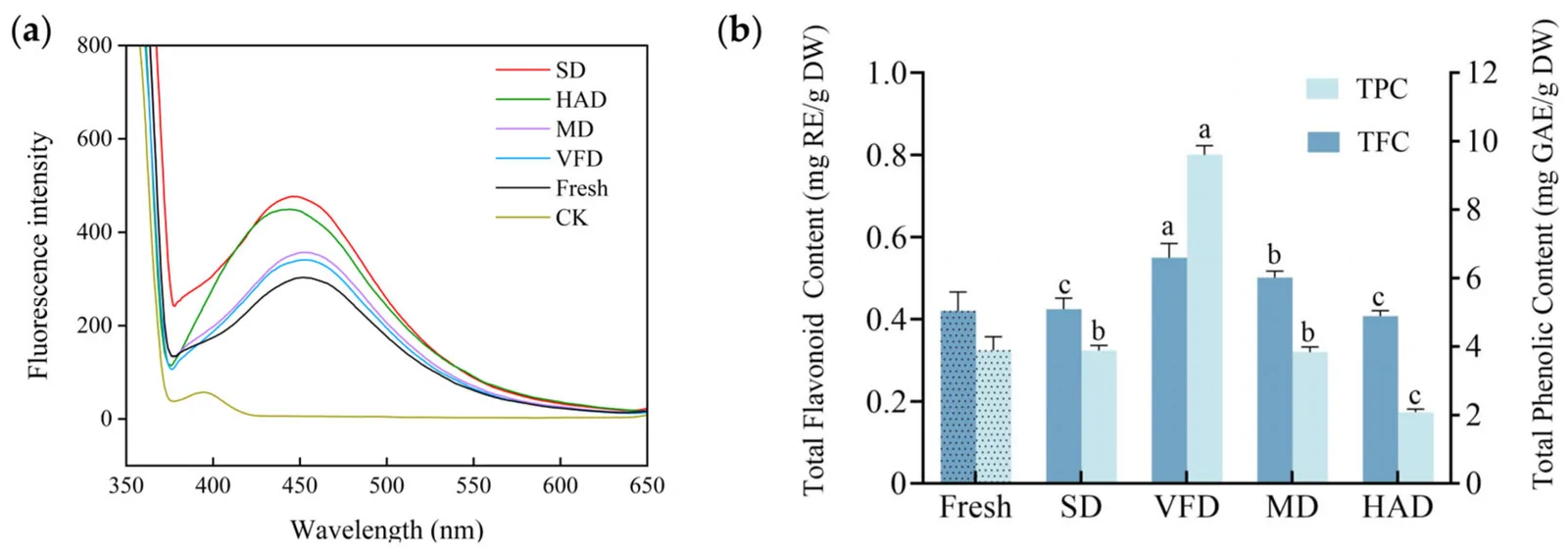
Effects of different drying methods on characteristic fluorescence spectra of Maillard reaction products, total polyphenol content and total flavonoid content of EI-AD. (**a**) Characteristic fluorescence spectra of Maillard reaction products, without statistical significance comparison; (**b**) Total polyphenol content and total flavonoid content. The fresh sample value (converted to DW basis) is shown as a reference baseline and was not included in the statistical comparison among drying treatments. Statistical comparisons are performed across groups on the horizontal axis, and different lowercase letters indicate significant differences according to Duncan’s test (*p* < 0.05).

**Figure 6:**
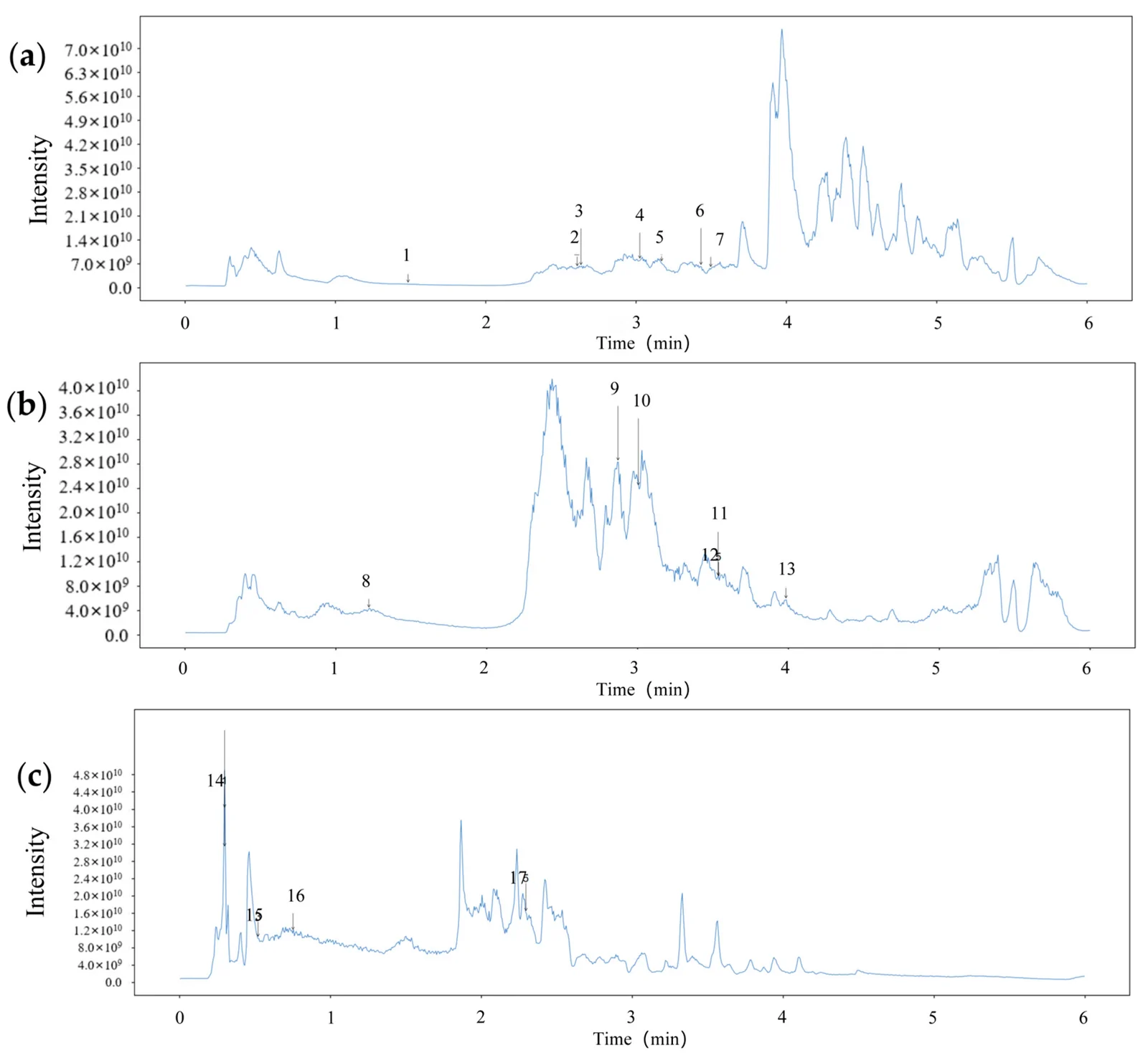
Total ion current chromatograms of EI-AD phenolic compounds under different drying methods. (**a**) RP-POS; (**b**) RP-NEG; (**c**) Amide-NEG.

**Figure 7:**
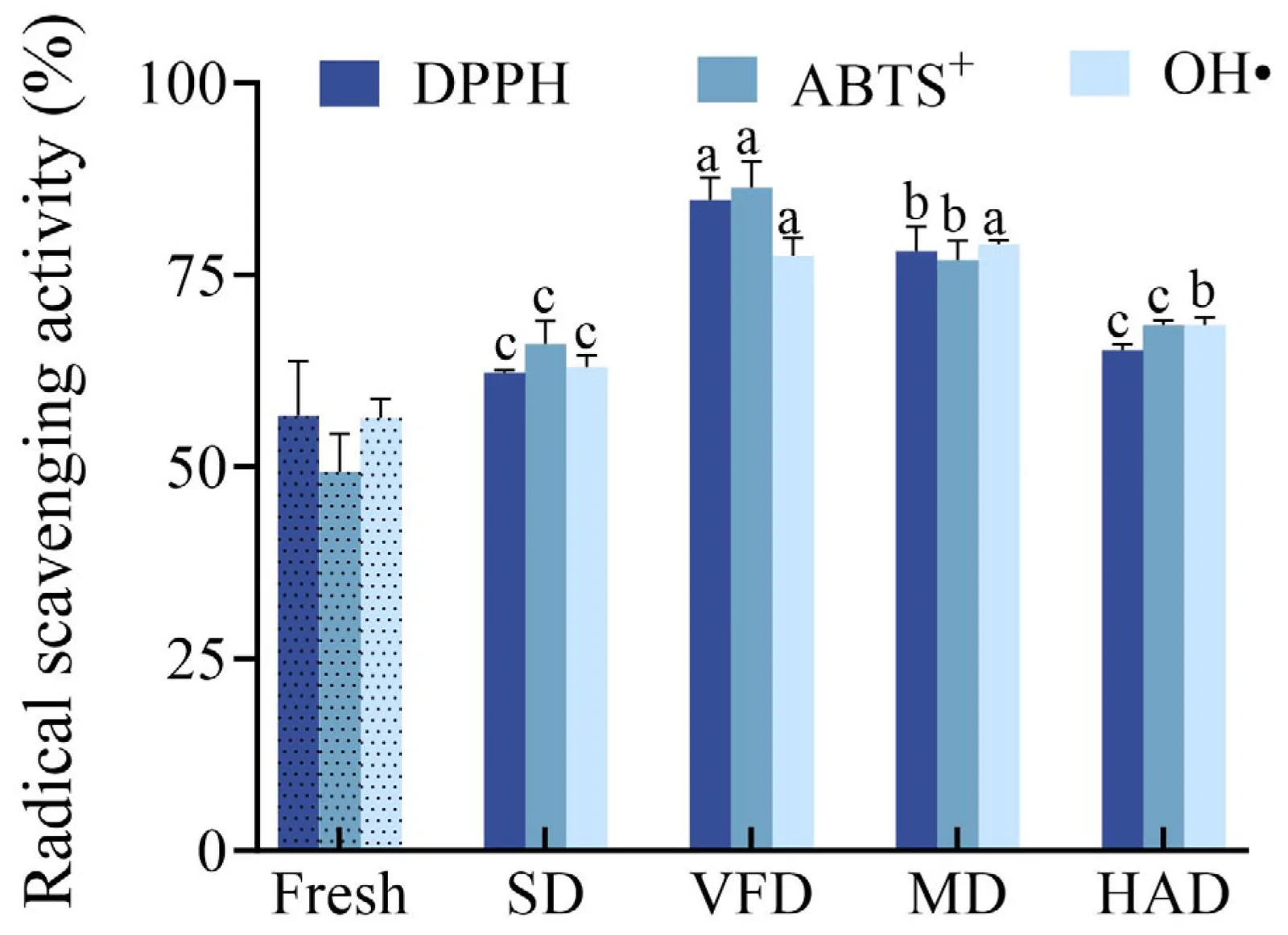
Effects of different drying methods on antioxidant activity in EI-AD. The fresh sample value (converted to DW basis) is shown as a reference baseline and was not included in the statistical comparison among drying treatments. Statistical comparisons are performed across groups on the horizontal axis. According to Duncan’s test, different lowercase letters indicate significant differences (*p* < 0.05).

**Figure 8:**
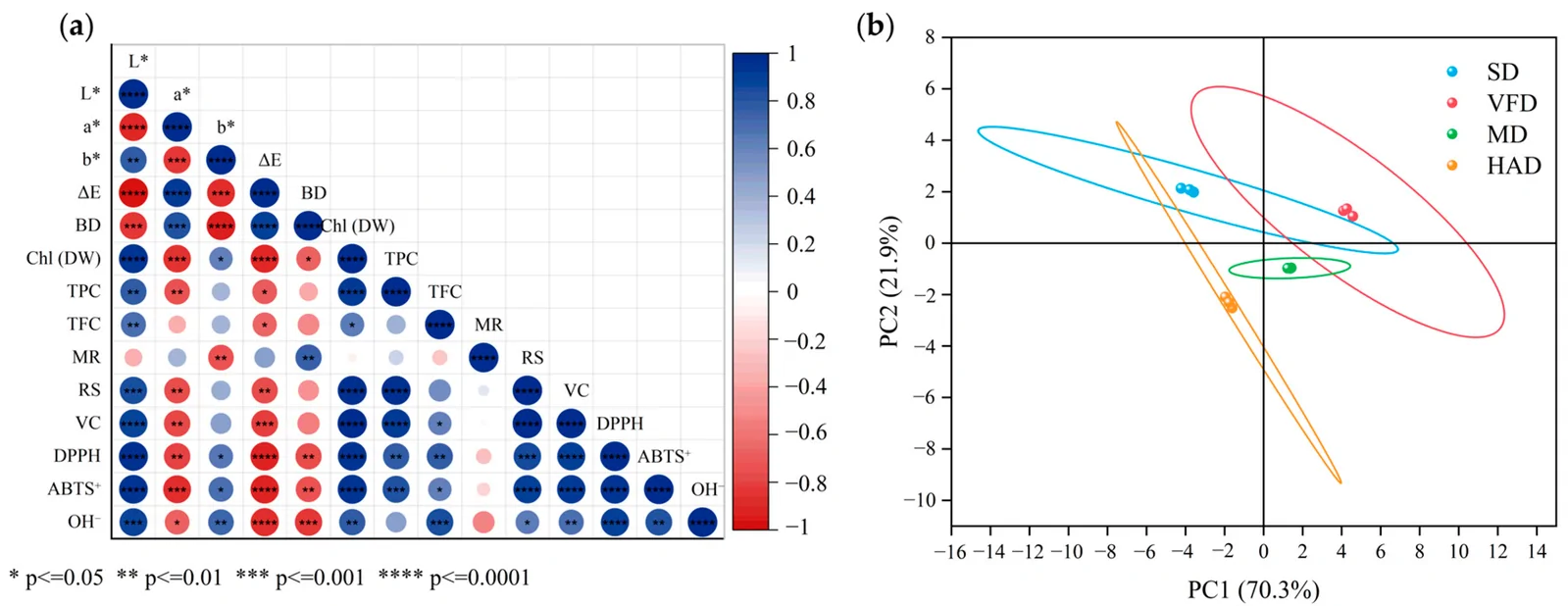
Correlation and PCA of quality parameters of EI-AD under different drying methods. (**a**) The correlation heatmap shows the Pearson correlation coefficients among quality parameters, bioactive components, and antioxidant activity of EI-AD under different drying methods. (**b**) PCA score plot of quality parameters of EI-AD under different drying methods.

**Table 1:** Effects of different drying methods on the color, browning degree, and chlorophyll retention rate of EI-AD.

Drying Method	L*	a*	b*	ΔE	Browning Degree (A/g)	Chlorophyll Content (%)
Fresh	40.61 ± 0.36 ^a^	−12.09 ± 0.33 ^d^	21.70 ± 0.41 ^a^	-	-	99.67 ± 0.58 ^a^
SD	18.00 ± 0.81 ^d^	−2.82 ± 0.12 ^a^	7.85 ± 0.81 ^d^	29.01 ± 1.09 ^a^	2.152 ± 0.003 ^a^	6.94 ± 0.57 ^d^
VFD	41.19 ± 0.71 ^a^	−8.56 ± 0.50 ^c^	17.87 ± 0.28 ^b^	5.88 ± 0.58 ^d^	0.212 ± 0.001 ^d^	62.17 ± 0.43 ^b^
MD	32.74 ± 0.82 ^b^	−5.16 ± 0.24 ^b^	15.38 ± 0.54 ^c^	13.22 ± 0.13 ^c^	0.449 ± 0.036 ^c^	31.47 ± 0.82 ^c^
HAD	23.95 ± 0.58 ^c^	−5.34 ± 0.08 ^b^	16.07 ± 1.04 ^bc^	19.78 ± 0.79 ^b^	0.679 ± 0.031 ^b^	7.16 ± 0.64 ^d^

Note: Results are expressed as mean ± standard error (SE). Statistical comparisons are performed by column. Means within the same column followed by different letters (a–d) indicate significant statistical differences among drying methods, as determined by one-way analysis of variance (ANOVA) and Duncan’s multiple range test.

**Table 2:** Relative MS Response of Phenolic Compounds in EI-AD Under Different Drying Methods.

Number	Compound Name	Type	Class	*m*/*z*	SD /Counts/CPS	VFD /Counts/CPS	MD /Counts/CPS	HAD /Counts/CPS
1	Chlorogenic acid	POS	Phenolic acids	355.1020	195,662,223.00	313,466,765.40	264,568,799.80	221,236,678.00
2	Ferulic acid	POS	Phenolic acids	195.0649	98,522,755.03	198,775,914.80	137,305,390.20	2,382,636.87
3	Vanillin	POS	Phenolic acids	153.0544	11,261,100.93	17,395,245.37	28,048,083.13	9,070,242.81
4	Hyperoside	POS	Flavonoids	465.1025	24,294,976.05	123,542,736.50	115,785,881.80	112,936,062.80
5	Isookanin	POS	Flavonoids	289.0705	4,613,075.25	53,651,759.77	44,886,917.12	26,885,344.70
6	Quercetin	POS	Flavonoids	303.0497	2,229,579.69	77,498,660.26	81,687,914.55	71,951,734.29
7	Hesperetin	POS	Flavonoids	303.0861	3,663,058.82	63,585,176.68	65,952,118.33	60,571,566.86
8	Pyrocatechol	NEG	Phenolic acids	109.0295	10,017,062.70	147,282,141.20	6,733,038.46	9,161,563.88
9	4-Hydroxycinnamic acid	NEG	Phenolic acids	163.0400	306,340,018.30	431,948,495.30	372,823,287.60	181,826,260.50
10	Rutin	NEG	Flavonoids	609.1458	204,474,321.50	437,230,291.60	433,208,254.60	408,925,575.10
11	Naringenin	NEG	Flavonoids	271.0611	1,761,476.07	29,246,299.81	30,356,404.35	27,361,665.32
12	3,5-Dicaffeoylquinic acid	NEG	Phenolic acids	515.1192	1,011,739,041.00	8,026,494,032.00	2,880,301,947.00	2,814,215,353.00
13	Kaempferol	NEG	Flavonoids	285.0404	88,591,154.20	93,055,233.01	126,963,248.60	90,342,784.73
14	Salicylic acid	NEG	Phenolic acids	137.0241	12,642,207.97	24,216,410.80	19,751,688.66	4,044,461.53
15	Okanin	NEG	Flavonoids	287.0556	564,268.58	32,310,113.97	21,079,212.05	12,674,082.62
16	Benzoic acid	NEG	Phenolic acids	121.0292	9,532,918.10	36,770,094.63	15,067,485.70	6,852,273.69
17	Caffeic acid	NEG	Phenolic acids	179.0347	286,509,174.30	437,252,859.90	290,420,293.00	211,150,768.30

Note: Statistical comparisons are performed by column.

## Data Availability

The original contributions presented in this study are included in the article. Further inquiries can be directed to the corresponding author.

## Abbreviations

The following abbreviations are used in this manuscript:

SD	Shade drying
VFD	Vacuum freeze-drying
MD	Microwave drying
HAD	Hot-air drying
EI-AD	Enzyme-inactivated *A. dracunculus*
PPO	Polyphenol oxidase
POD	peroxidase
BD	Browning degree
Chl	Total chlorophyll content
FTIR	Fourier-transform infrared
TFC	Total flavonoid content
TPC	Total phenolic content
VC	Vitamin C
RS	Reducing sugar
MR	Maillard reaction products
NSA	Nitrite scavenging capacity
